# Body Fat Distribution and Insulin Resistance

**DOI:** 10.3390/nu5062019

**Published:** 2013-06-05

**Authors:** Pavankumar Patel, Nicola Abate

**Affiliations:** Division of Endocrinology, Department of Internal Medicine, University of Texas Medical Branch, 301 University Blvd, Galveston, TX 77555, USA; E-Mail: niabate@utmb.edu

**Keywords:** adipose tissue, adipose tissue distribution, adipose tissue dysfunction, adipose tissue inflammation, body fat distribution, insulin resistance

## Abstract

The burden of obesity has increased globally over the last few decades and its association with insulin resistance and related cardio-metabolic problems have adversely affected our ability to reduce population morbidity and mortality. Traditionally, adipose tissue in the visceral fat depot has been considered a major culprit in the development of insulin resistance. However, there is a growing body of evidence supporting the role of subcutaneous truncal/abdominal adipose tissue in the development of insulin resistance. There are significant differences in the functional characteristics of subcutaneous abdominal/truncal *vs.* intraabdominal *vs.* gluteo-femoral fat depots. More recently, mounting evidence has been supporting the role of adipose tissue function in the development of metabolic complications independent of adipose tissue volume or distribution. Decreased capacity for adipocyte differentiation and angiogenesis along with adipocyte hypertrophy can trigger a vicious cycle of inflammation leading to subcutaneous adipose tissue dysfunction and ectopic fat deposition. Therapeutic lifestyle change continues to be the most important intervention in clinical practice to improve adipose tissue function and avoid development of insulin resistance and related cardio-metabolic complications.

## 1. Introduction

The increasing global burden of obesity has become a major public health problem. The most recent (2010) analysis by the International Association for the Study of Obesity (IASO) and International Obesity Taskforce (IOTF) reports that the number of adults globally who are overweight or obese is one billion and 475 million, respectively [1]. IASO/IOTF also estimate that the number of children worldwide who are either overweight or obese is 200 million [1]. Similar trends have been noticed in the United States. The National Health and Nutrition Examination Survey (NHANES) estimates that 33.8% of adults (age 20 years or more) and 16.8% of children and adolescents (age 2–19 years) are obese [2,3]. Obesity is associated with a variety of medical conditions including cardiovascular disease (CVD), diabetes mellitus, hypertension, dyslipidemia, nonalcoholic fatty liver disease, cancers, and sleep apnea [4].

Insulin resistance is an underlying key pathophysiologic process in the development of cardio-metabolic disorders among obese individuals. Insulin resistance leads to development of an atherogenic dyslipidemic profile, and prothrombotic and proinflammatory states. Dyslipidemia in insulin resistant individuals is characterized by elevated triglycerides, apolipoprotein B, small dense low density lipoprotein (LDL) particles, and reduced high density lipoprotein (HDL) concentration and smaller HDL particle size. Insulin resistance also leads to elevated blood pressure and glucose intolerance, which in the presence of genetic and environmental factors, can progress to hypertension and type 2 diabetes mellitus [5].

The relationship between obesity and cardio-metabolic disorders is more complex than it appears. Many epidemiologic and clinical studies have suggested that 10%–40% of obese individuals are metabolically healthy. The metabolically healthy obese (MHO) phenotype exhibits higher insulin sensitivity, absence of hypertension, and favorable lipid, inflammation, hormonal and liver enzyme profile [6]. Vice versa, metabolic abnormalities can occur in normal weight individuals. This metabolically obese but normal weight phenotype is characterized by not being obese on the basis of height and weight but with hyperinsulinemia, insulin resistance and increased risk for type 2 diabetes, hypertriglyceridemia and atherosclerosis [7]. One explanation for metabolically obese normal weight profile is variability in the body fat content for any given Body Mass Index (BMI). Recent studies performed within the NHANES III population have revealed that about 10% of the US population is estimated to have a normal BMI but increased body fat content [8,9].

However, another major contributing factor in variability in body fat-related metabolic complications is fat distribution in various adipose tissue areas. Adipose tissue can be divided into truncal region or peripheral region. Truncal adipose tissue includes subcutaneous fat in thoracic and abdominal region and also fat in intrathoracic and intraabdominal regions. Peripheral adipose tissue includes subcutaneous depots in upper and lower extremities [10]. 

The association between regional fat distribution and cardio-metabolic complications was first suggested by Vague in 1947. Vague described two patterns of adipose tissue distribution—android (upper body) and gynoid (lower body)—and suggested that android obesity was associated with diabetes, coronary artery disease, gout and uric acid renal stones [10]. Later on in 1980s, Lapidus L *et al.* reported that Waist to Hip circumference ratio, compared to other anthropometric measures, was positively associated with a 12-year incidence of myocardial infarction, angina pectoris, stroke, and death [11]. Subsequently, many clinical studies have established association between increased Waist to Hip circumference ratio and cardiovascular diseases. The INTERHEART study examined the effect of different measures of obesity on the risk of myocardial infarction in multiple populations from 52 countries across the world [12]. Study investigators found that Waist to Hip ratio, and the waist and the hip circumferences, were highly associated with risk of acute myocardial infarction independent of other risk factors. The European Prospective Investigation into Cancer and Nutrition in Norfolk Cohort (EPIC) study prospectively examined the relationship between body fat distribution and risk of coronary heart disease [13]. After 9.1 years of follow up, investigators reported that waist circumference was a significant estimate for coronary artery disease events. In the Atherosclerosis Risk in Communities (ARIC) study, Waist to Hip ratio was associated with increased risk for non-lacunar and cardio-embolic stroke [14]. Waist to Hip ratio, being a simple index of body fat distribution, when elevated crudely indicates increased proportion of abdominal adipose tissue. In fact, over the last few decades evidence has accumulated to support the concept of abdominal adipose tissue as a determinant of insulin resistance.

To be more precise, many investigators have reported that intraabdominal (visceral) adipose tissue is a major contributor to metabolic risk [15,16,17], whereas some investigators have suggested that subcutaneous adipose tissue may have a protective role [18]. Intraabdominal adipose tissue has increased metabolic (both lipogenesis and lipolysis) activity. According to the portal vein hypothesis, free fatty acids, as a product of lipolysis, directly enter the liver via portal vein and lead to increased lipid synthesis, gluconeogenesis, and insulin resistance. This can result in hyperlipidemia, glucose intolerance, hypertension and ultimately atherosclerosis [15]. Excess free fatty acids can inhibit skeletal muscle glucose uptake and lead to peripheral insulin resistance [10].

However, if visceral fat was a major contributor to metabolic risk, visceral adipose tissue should be the major source of systemic free fatty acid flux. Only a small portion of total body fat, 15%–18% in men and 7%–8% in women, is located in the abdominal cavity [19]. Visceral fat contributes to only 15% of the total systemic free fatty acids whereas majority of free fatty acid is contributed by nonsplanchnic adipose tissue [10,20]. This observation raises the doubt over notion that visceral adipose tissue is a sole determinant of peripheral insulin sensitivity. 

We have examined the relationships between generalized and regional adiposity and insulin sensitivity in a group of nondiabetic men with varying degree of obesity [21]. We concluded that subcutaneous truncal fat plays a major role in obesity related insulin resistance in comparison to visceral or retroperitoneal fat. Subsequently, we examined a similar relationship among men with noninsulin dependent diabetes mellitus (NIDDM) and found that NIDDM men had a fat distribution pattern that favors truncal subcutaneous depot than peripheral subcutaneous or intraperitoneal fat depot [22]. Truncal subcutaneous fat content had a stronger correlation with insulin sensitivity than visceral fat among NIDDM men. Goodpaster, B.H. *et al.* has also demonstrated a stronger relationship between subcutaneous abdominal fat and insulin sensitivity [23]. Cross sectional analyses of data from the Amsterdam Growth and Health Longitudinal Study by Ferreira I *et al.* revealed that high subcutaneous trunk fat was associated with arterial stiffness [24]. The stronger relationship between subcutaneous adipose tissue and insulin resistance can be related to relatively larger subcutaneous adipose tissue mass. Subcutaneous abdominal fat mass is two times more than the intraperitoneal fat mass. However, if we consider total subcutaneous truncal fat mass, it is approximately 4–5 times more than the intraperitoneal fat mass [21,22,25]. Similarly in women, subcutaneous abdominal fat area at a L4–L5 level is approximately five times more than the visceral fat area at the same level [25,26,27]. We can postulate that subcutaneous truncal adipose tissue, merely because of its larger volume, can be a major contributor of systemic free fatty acid flux and therefore, insulin resistance.

Inflammation in adipose tissue, as suggested by the presence of macrophage in the form of crown-like structures (CLS), has been identified as a mediator of systemic insulin resistance. Apovian, C.M. *et al.* examined relationships between adipose tissue macrophage infiltration and insulin resistance and vascular endothelial dysfunction in obese individuals [28]. Inflamed subcutaneous adipose tissue phenotype, characterized by the presence of macrophage in crown-like structures, was associated with systemic hyperinsulinemia, insulin resistance, impaired endothelium-dependent flow-mediated vasodilatation and elevated plasma hs-CRP levels. Other investigators have also reported similar association between macrophage infiltration of subcutaneous adipose tissue and insulin resistance and low grade systemic inflammation [29,30]. Lê, K.A. *et al.* examined the influence of subcutaneous adipose tissue (SAT) inflammation on hepatic fat fraction, visceral adipose tissue, insulin sensitivity, beta cell function and SAT gene expression [30]. Subcutaneous adipose tissue inflammation, independent of total adiposity, was associated with partitioning of fat towards the visceral adipose tissue and liver and altered beta cell function. In addition, several genes belonging to nuclear factor-κB stress pathway were up regulated suggesting stimulation of inflammatory mediators.

Attempts have been made to identify triggers for adipose tissue inflammation and subsequent development of insulin resistance. In the presence of increased caloric intake, adipocytes in subcutaneous adipose tissue with decreased adipogenic potential undergo hypertrophy. This results in local hypoxic environment and macrophage infiltration. van Tienen, F.H. *et al.* reported that preadipocytes of type 2 diabetes subjects display decreased expression of genes involved in differentiation suggestive of decreased adipogenesis [31]. Goedecke, J.H. *et al.* reported association between decreased insulin sensitivity and decreased expression of adipogenic and lipogenic genes in subcutaneous adipose tissue of obese black South African women [32]. Lundgren, M. *et al.* examined the relationship between fat cell size and insulin sensitivity [33]. Enlarged adipocytes were found in patients with type 2 diabetes and prediabetic individuals and were an independent marker of insulin resistance in prediabetic subcutaneous adipose tissue. We have reported that migrant South Asians, compared to Caucasians, have excess insulin resistance without increase in the intraperitoneal fat mass. They had increased adipocyte size in the subcutaneous abdominal adipose tissue and elevated plasma hs-CRP suggestive of low grade systemic inflammation [34,35]. In addition to decreased adipogenesis, subcutaneous adipose tissue exhibits decreased angiogenic capacity. Gealekman, O. *et al.* reported that angiogenic capacity of subcutaneous abdominal adipose tissue decreased with increasing body mass index but it did not change in visceral adipose tissue [36]. Decrease in angiogenic capacity correlated with insulin resistance, which suggests that impairment in subcutaneous adipose tissue angiogenesis may contribute to metabolic complications.

In contrast to abdominal subcutaneous adipose tissue, larger subcutaneous thigh fat has a protective effect. The Health, Aging, and Body Composition Study reported that large subcutaneous thigh fat was independently associated with more favorable glucose (in men) and lipid profile (in both genders) [37]. The Australian Diabetes, Obesity and Lifestyle Study, a large population based study, examined association between waist and hip circumferences to components of metabolic syndrome [38]. After adjustment for age, BMI and waist, a larger hip circumference was associated with a lower prevalence of undiagnosed diabetes and dyslipidemia. Association with undiagnosed hypertension was weaker. Vice versa, low subcutaneous thigh fat was associated with the adverse metabolic profile. The Quebec family study examined effects of waist and hip circumferences on cardiovascular risk factors [39]. A narrow hip circumference (adjusted for age, BMI, and waist circumference) was associated with low HDL-cholesterol and high glucose concentrations in men and high triacylglycerol and insulin concentrations in men and women.

Finally, it is worth summarizing the role of subcutaneous adipose tissue characteristics in the development of insulin resistance. It can be envisioned that persisting excess in caloric intake is usually met by an increase in fat deposition, predominantly occurring in the subcutaneous adipocytes. Truncal *vs.* peripheral deposition could be determined by genetic and environmental factors, currently a matter of intense investigation. This buffering function of subcutaneous adipose tissue can be envisioned as continuing until a tipping point is reached. This happens when decreased adipogenesis and decreased angiogenesis, along with adipocyte hypertrophy, sets up an inflammatory reaction characterized by activation of nuclear factor-KB pathway. The resultant effects are: down regulation of cellular insulin signaling, recruitment of additional macrophages through monocyte chemoattractant protein 1, propagation of inflammation by interleukins and tumor necrosis factor alpha, and tissue matrix remodeling through matrix metalloproteinase-9 [30]. These inflammatory mediators, as well as yet not well-defined metabolic pathways, may limit the recruitment and maturation of new adipocytes. Subcutaneous adipose tissue, which is now dysfunctional and not able to expand, cannot continue to serve as an energy buffer. At this point, positive energy balance results in a spillover of fatty acids and triglycerides in ectopic tissues. Fat deposition in skeletal muscle results in decreased activity of glucose transporter (GLUT 4) and decreased glucose uptake. Hepatic steatosis results in hepatic insulin resistance which leads to decreased glucose uptake and increased glucose production. Free fatty acid accumulation in the heart can result in diastolic dysfunction [40]. It is possible that, depending on the environmental and genetic factors, “tipping point” may be reached at different levels of adiposity, including in a non-obese range, and it may not be reached at all even with morbid obesity. It is possible that increased fat in subcutaneous depot relative to visceral depot may not be accompanied by an increase in insulin resistance since “tipping point” is not reached.

## 2. Conclusion

There is enough evidence in the existing literature for association between increased adiposity and insulin resistance. Attempts have been made to identify one particular adipose tissue depot as a sole contributor to insulin resistance and cardio-metabolic complications. Many investigators have suggested that visceral adipose tissue is a major contributor to insulin resistance. Our previous studies, in concordance with those of other investigators, suggest that subcutaneous truncal adipose tissue has significant impact on the development of insulin resistance. In fact, subcutaneous adipose tissue appears to have a major function as a metabolic buffer. In the presence of positive caloric balance, subcutaneous adipose tissue eventually becomes dysfunctional due to adipocyte hypertrophy, decreased adipogenesis and angiogenesis. At this point, buffering function is lost and excess free fatty acids spill over into ectopic tissues, resulting in tissue specific consequences. Therapeutic lifestyle change, including physical activity, weight loss and healthy diet continue to be the most important interventions to restore the buffering capacity of subcutaneous adipose tissue. PPAR-gamma ligands have also been shown to serve the same purpose and decrease ectopic fat deposition. Future research will have to be focused on effective and safe therapeutic targets to accomplish the proven metabolic benefit of amelioration in adipose tissue function and ultimately allow more effective interventions to improve morbidity and mortality among metabolically obese patients.
